# Do in-person nationwide university entrance exams affect COVID-19 transmission? An experience from Iran

**DOI:** 10.1186/s12889-025-23741-w

**Published:** 2025-08-02

**Authors:** Alireza Mirahmadizadeh, Zahra Mehdipournamdar, Zahra Jaafari, Abdolrasoul Hemmati, Ata Miyar, Mohammad Hossein Sharifi

**Affiliations:** 1 Non-communicable Diseases Research Center, Shiraz University of Medical Sciences, Shiraz, Iran; 2 Student Research Committee, Shiraz University of Medical Sciences, Shiraz, Iran; 3 Health Affairs, Shiraz University of Medical Sciences, Shiraz, Iran; 4 Schulich School of Medicine and Dentistry, University of Western Ontario (Western University), London, ON Canada; 5 Research Center for Traditional Medicine and History of Medicine, Shiraz University of Medical Sciences, Shiraz, Iran

**Keywords:** COVID-19, In-person university exams, Pandemics

## Abstract

**Background:**

Nationwide University Entrance Exams (NUEEs) are essential scheduled exams that, in some countries, have been conducted in-person even during the COVID-19 pandemic. Considering the potentially fatal consequences of any pandemic-related decisions by policymakers, the response of the national health systems and regulatory bodies to this matter must be evidence-based. This study has been the first to evaluate the effects of NUEEs on measurements and indices associated with COVID-19.

**Methods:**

Five NUEEs were conducted in the year 2020 from July 30 to August 22 in the province of Fars, located in the south of Iran. The trends of emergency services, 16-hour special health center visits, PCR tests, hospital services, and death rates due to COVID-19 following these NUEEs were assessed in a 72-day span in this study.

**Results:**

All COVID-19 related indices and measurements in this study showed a decreasing trend across the board with the exception of the number of total and positive polymerase chain reaction ( PCR) tests.

**Conclusion:**

This study found that following the conduction of 2020 NUEEs, there was no increasing trend in the number of COVID-19 cases and the associated indices and measurements. The findings of this study will be valuable for future practices, health protocols, and policies. This study also highlighted the importance of the constant implementation of strict health protocols and measures before, during, and after each NUEE.

## Background

Following the outbreak of Coronavirus Disease 2019 (COVID-19), according to reports from the World Health Organization (WHO), up until September 14th, 2020, Iran had the second number of confirmed cases of COVID-19 (402,029) among Eastern Mediterranean countries [1]. Fars, one of the largest provinces of Iran, had been highly affected by COVID-19 and was responsible for a significant portion (47,988) of the total numbers of confirmed cases of COVID-19 in Iran up until September 2020 [1]. Since the occurrence of the first case of COVID-19 in Iran on February 19, 2020, until February 23, a total of 43 cases of this disease were reported, eight of whom resulted in death [2].

The COVID-19 pandemic has significantly damaged education [3]. In this regard, UNESCO has published a report that states that 172 countries closed schools completely by April 2020, and a high percentage of all students in the world (84.8%) were affected [4]. In some countries including the United States, in-person classes were stopped and campuses were closed [5, 6]. Many students and lecturers were forced to learning and teaching remotely, respectively [7]. Among other countries affected by this pandemic, Sweden, Hong Kong, Indonesia, Myanmar, South Korea, and China can be mentioned. According to a report published by UNESCO in April 2020, it should be noted that the mentioned countries postponed their nationwide exams due to the widespread prevalence of the disease [8].

Indoor gatherings, including in-person academic classes, have been shown to increase the risk of COVID-19 transmission [9–12]. However, there is not sufficient evidence of the effect of Nationwide University Entrance Exams (NUEEs), which can be considered large-scale public gatherings, on COVID-19 transmission. NUEEs are essential scheduled exams that, in some countries, have been conducted in-person even during the recent pandemic. Evidently, Iran was one of the first countries that restarted their NUEEs with the participation of more than 1,500,000 candidates in 2020. India, despite having nearly 4 million cases, and Vienna were also among the first regions to proceed with in-person nationwide exams [13, 14]. For example, conducting examinations in India due to the lack of preparation from exam authorities for students in this Covid-19 pandemic has finally made many fail their exam [15]. To reduce the possibility of transmission of COVID-19 during NUEEs, various health protocols were utilized by different countries. Iran’s special health protocols included wearing masks at all times, regular hand washing or use of hand sanitizers, and disinfecting surfaces per the instructions of the Ministry of Health [16].

Evaluating the effects of NUEEs on COVID-19 transmission, hospitalization, and associating indices and measurements would be crucial for future policies and regulations, including the determination of health protocols, crisis management, support for health services, and education and awareness. Given the potentially fatal consequences of pandemic-related decisions, it is essential that policymakers base their responses on evidence. This study evaluates the impact of NUEEs on COVID-19 transmission. Factors such as the burden on emergency and hospital services, and changes in death rates following the NUEEs in the province of Fars in Iran in a 72-day span (almost twice the contagious period of COVID-19), which includes the period before and after the NUEEs were analyzed. The results would help policymakers establish better policies and decisions in the future with regards to in-person nationwide and universal exams during pandemics and similar situations.

In this study, we answer the following questions:

1. How does NUEEs participation influence COVID-19 outcomes (infection rates, hospital admissions, and mortality) among individuals visiting health centers?
2. What are the trends in emergency service metrics (including hospital occupancy rates and PCR testing) during the pandemic, and how do they correlate with NUEEs participation?

## Methods

### Design and setting of the study

This 72-day *observational study* was conducted from July 5 to September 14, 2020, in the province of Fars, located in the south of Iran. Fars has a population of 4,871,274, of whom 120,366 attended five different types of NUEEs since the outbreak of COVID-19 including exams for admission to both undergraduate and postgraduate programs (Master’s and PhDs in general and medical sciences).

The trends of emergency services, 16-hour special health center visits, PCR tests, hospital services, and death rates due to COVID-19 following these NUEEs were assessed in this study. We evaluated the impact of in-person nationwide university exams among 280 individuals of all people who had visited health centers or hospitals and had a history of NUEE participation either by themselves or a close family member, on changes in the following COVID-19-related indices and measurements from 26 days before to 24 days after these exams:

- Daily number of consults with Emergency Medicine Services (EMS),
- Daily number of EMS trips related to COVID-19,
- Daily number of patients transferred by Emergency Medical Services (EMS),
- Daily number of visits to 16-hour special healthcare centers for COVID-19,
- Daily number of referrals from 16-hour special healthcare centers for COVID-19 to hospitals,
- Daily number of Polymerase Chain Reaction (PCR) tests performed on patients with Severe Acute Respiratory Syndrome Coronavirus-2 (SARS-CoV-2),
- Daily number of positive PCR tests,
- Daily COVID-19 case rates (per 1,000,000),
- Daily COVID-19 death rates (per 1,000,000),
- Daily number of total hospital admissions,
- Daily hospital occupancy rates for intensive care units (ICUs) because of COVID-19, and.
- Daily hospital occupancy rates for non- intensive care unit (ICU) COVID-19 admissions.

We also assessed the individual characteristics of the NUEEs’participants. For this purpose, according to the guidelines issued to hospitals and health centers, personal and related information of individuals referred to hospitals (all hospitals: 18) and health centers (all health centers: 45) because of being suspected of having COVID-19 were registered in an internet-based system. Registration was performed by trained healthcare workers. SARS-CoV-2 PCR test was performed on the participants who gave a positive answer to one or both of the following questions:

- Did you take any of the NUEEs?
- Did any of your family members take any of the NUEEs?

The NUEEs participants with positive SARS-CoV-2 PCR tests were considered confirmed cases and were closely monitored for 21 days to detect any signs or symptoms of COVID-19.

All data were gathered from CORONALAB, CORONA dashboard indices, EMS database, Medical Care Monitoring Center (MCMC), Hospital Information Systems (HIS), and National Organization of Educational Testing for examiner data. The information about the data sources, types of data collected and how they are collected is shown in Table 1.

**Table 1:** Information about the data sources, types of data collected, and how they are collected

Data sources	Types of data collected	How they are collected
Medical Care Monitoring Center (MCMC)	• Online monitoring of all hospitals • Patient identification data, phone number, and mailing address; Admission data; The name of the hospital; Early signs and symptoms of the disease; CT Scan; PaO2 (saturation), Underlying conditions and comorbidity); COVID-19 test result; Intubation and admission to intensive care unit (ICU); Outcome; Date of discharge or death	According to the Code of Ethics for Study and Official Correspondence
Hospital Information Systems	• Data elements related to COVID-19 • Date of discharge • Date of hospital admission • Comorbidity	According to the Code of Ethics for Study and Official Correspondence
Coronalab	• PCR test results	According to the Code of Ethics for Study and Official Correspondence
Dashboard	• Morbidity and mortality indices • Prevalence and incidence related to COVID-19 • COVID-19 test result • Intubation, admission to intensive care unit (ICU) • Outcome • Date of discharge or death	According to the Code of Ethics for Study and Official Correspondence
Emergency Medical Services (EMS)	• Calls related to Coronavirus disease through the 115 system • The flow of definitive patient information in the MCMC system	According to the Code of Ethics for Study and Official Correspondence
National Organization of Educational Testing	• Number of participants in the exam • Location of exam	According to the Code of Ethics for Study and Official Correspondence

### Statistical analysis

In this study, 26 days before, 12 days during, and 24 days after these exams were considered to evaluate the changes in the trends of the aforementioned measurements. The analysis used was a linear regression (significance level of 0.05) in which the outcome variable over time was the aforementioned indicators of COVID-19, which, using a coefficient, showed an upward or downward trend. Data analysis was done using SPSS version 21.0 (IBM, SPSS, Chicago, IL, USA).

## Results

The exact date and duration of monitoring for each NUEE are provided in Table 2.

**Table 2:** Exact date and the duration of monitoring for each nationwide university entrance exam (NUEE)

Type of exam	Date of exam	Duration of monitoring	Number of participants in each NUEE	Positive PCR results
PhD (non-medical sciences)	30/7/2020	30/7/2020 to 21/8/2020	8,750	8
PhD (medical sciences)	31/7/2020–2/8/2020	31/7/2020 to 23/8/2020	3,230	7
Master’s (non-medical sciences)	6/8/2020–7/8/2020	6/8/2020 to 28/8/2020	18,340	12
Master’s (medical sciences)	13/8/2020–14/8/2020	13/8/2020 to 4/9/2020	9,014	16
Undergraduate university admission	19/8/2020–22/8/2020	19/8/2020 to 14/9/2020	81,032	50
Total duration of monitoring	**-**	30/7/2020 to 14/9/2020	120,366	93

During the 72-day period of our study, 29,744 new cases of COVID-19 were identified in the province of Fars. Throughout the study period, 280 individuals of all people who had visited health centers or hospitals had a history of NUEE participation either by themselves or a close family member. 143 of the participants were male (51.1%) and the mean age of all participants was 38.8 ± 1.86 years. Overall, 93 (33%) had a positive PCR results. Out of 280 individuals, 199 visited 16-hour special health centers for COVID-19, while the remaining 81 (28.9%) were referred to hospitals. Notably, 96 individuals (34.6%) had a positive history of close contact with individuals suspected of COVID-19 and 27 of them (9.1%) stated that they had traveled within the last 14 days.

To evaluate changes in the COVID-19 pandemic in relation to the NUEEs, five key indices including emergency services, 16-hour special health center visits, number of PCR tests, hospital services, and death rates due to COVID-19 were evaluated. The detailed results are provided in Table 3.

**Table 3:** Linear regression results for each COVID-19 associating measurements

Category	Measurement	Coefficient	95% Confidence Interval (CI)	*P*-value	Trend
Emergency Services for suspected COVID-19 Patients	Consults (No.)	−4.47	−4.89, −4.05	< 0.001	Decreasing
	Trips (No.)	−0.70	−0.80, −0.61	< 0.001	Decreasing
	Transfers (No.)	−0.17	−0.21. −0.13	< 0.001	Decreasing
16-hour special health centers services for COVID-19	Visits (No.)	−5.95	−8.66, −3.24	< 0.001	Decreasing
	Referrals (No.)	−0.38	−0.48, −0.28	< 0.001	Decreasing
PCR tests	Total tests (No.)	−17.14	−20.8, −13.48	< 0.001	Decreasing
	Positive tests (No.)	− 0.5.9	− 0.7.10, −4.71	< 0.001	Decreasing
	Percentage of positive tests	−0.62	−0.10, −0.02	< 0.001	Decreasing
Hospital services	Admission (No.)	−2.08	−2.41, −1.75	< 0.001	Decreasing
	Occupied non-ICU bed rate	−0.60	−0.74, −0.46	< 0.001	Decreasing
	Occupied ICU bed rate	−0.26	−0.31, −0.20	< 0.001	Decreasing
Daily incidence of death		−0.02	−0.02, −0.10	< 0.001	Decreasing

**Fig. 1:**
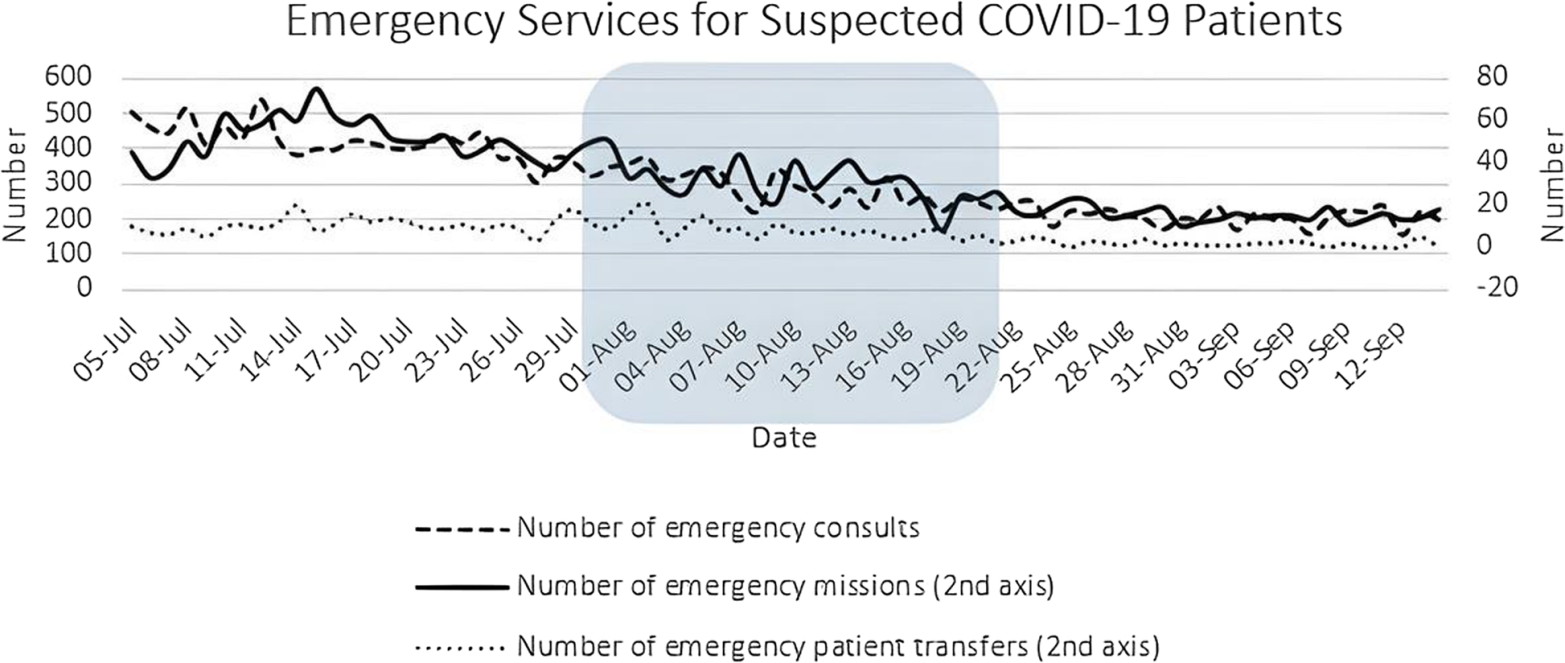
Number of emergency consults, trips, and transfers regarding suspected COVID-19 patients from July 5 to September 14, 2020. The square demonstrates the period in which NUEEs were conducted

The effect of NUEEs on the COVID-19-related measurement in terms of emergency services was assessed via linear regression analysis for three indices: the number of emergency telephone consults (coefficient: −04.47, CI: −4.89, −4.05), the number of outpatient trips (coefficient: −0.70, CI: −0.80, −0.61), and the number of transfers to hospitals (coefficient: −0.17, CI: −0.21, −0.13). As it is showed in Fig. 1, a decreasing trend prevailed across all components of this measurement during the study period (*P* < 0.05 for all sub-variables).

**Fig. 2:**
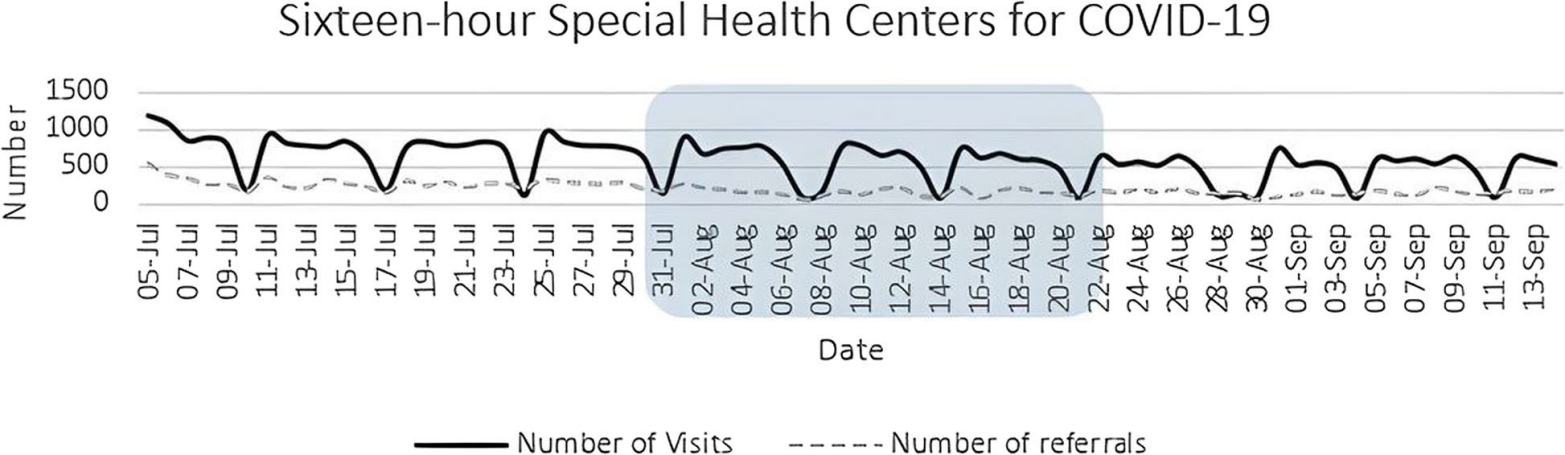
Number of daily visits and referrals to hospitals in 16-hour special health centers for COVID-19 from July 5 to September 14, 2020. The square demonstrates the period in which NUEEs were conducted

In the 16-hour special health centers for COVID-19, the number of visits (coefficient: − 0.5.95, 95% CI −8.66 to −3.24) and referrals to hospitals (coefficient: − 0.038, 95% CI −0.48 to −0.28) remained almost stable over the given period (*P* < 0.05 for both). The sudden decreases in the plots are attributable to the effect of off days on weekends (Fig. 2).

**Fig. 3:**
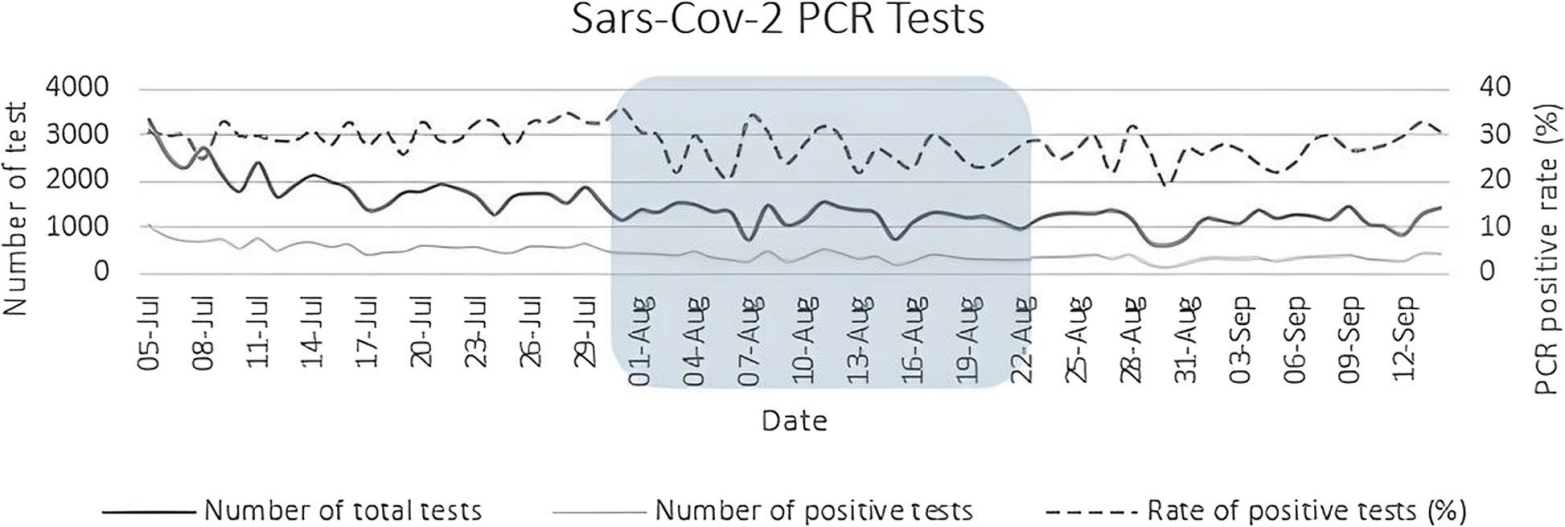
Trend of total number of performed and positive PCR tests from July 5 to September 14, 2020. The square demonstrates the period in which NUEEs were conducted

Regarding the number of total and positive PCR tests, the trend was neutral and the numbers did not show any increasing or decreasing trend, rather a fluctuation throughout the study period (*P* < 0.05). The number of total PCR tests fluctuated between 1000 and 3,500 tests (−17.14, CI: −20.8, −13.48). The highest percentage of positive PCR tests (36%) was recorded on August 10, 2020 (Fig. 3).

**Fig. 4:**
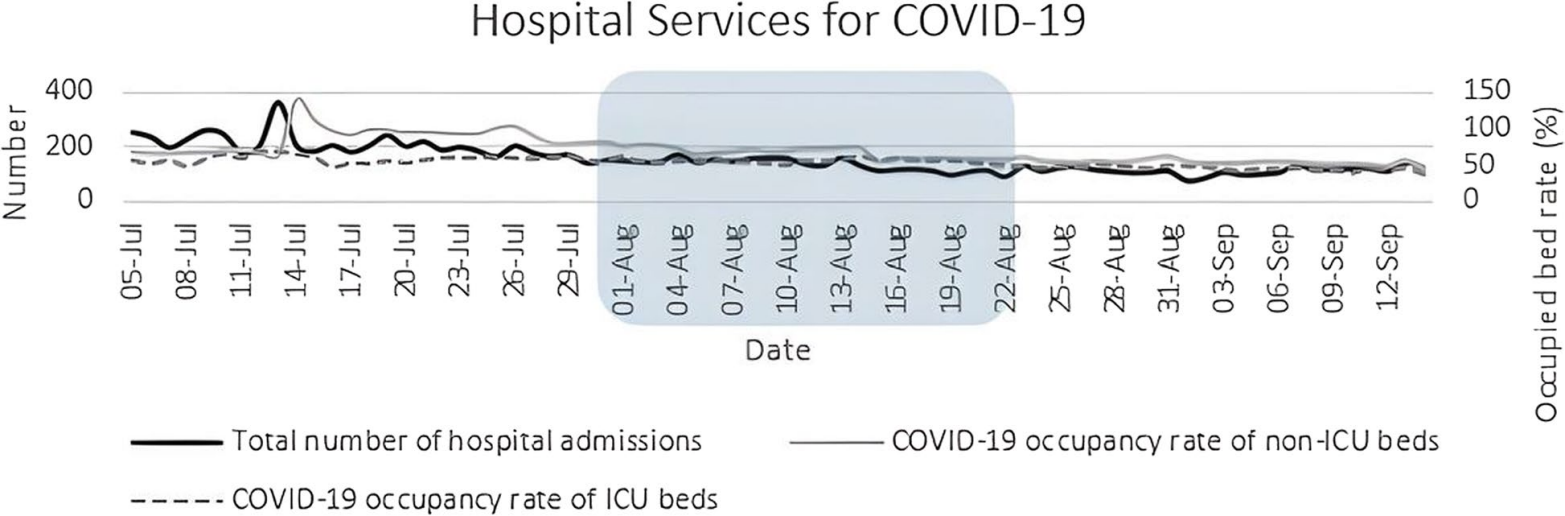
Number of total hospital admissions and occupancy rates of non-ICU and ICU beds due to COVID-19 from July 5 to September 14, 2020. The square demonstrates the period in which NUEEs were conducted

In terms of hospital statistics, a decreasing pattern was detected in the number of total admissions (coefficient: −2.08) and occupancy rates of non-ICU and ICU beds due to COVID-19 (coefficients: −0.6 and − 0.26, respectively). The maximum and minimum number of total admissions were 368 and 73, respectively (Fig. 4). The p-value was below 0.05 for all indices in this measurement.

**Fig. 5:**
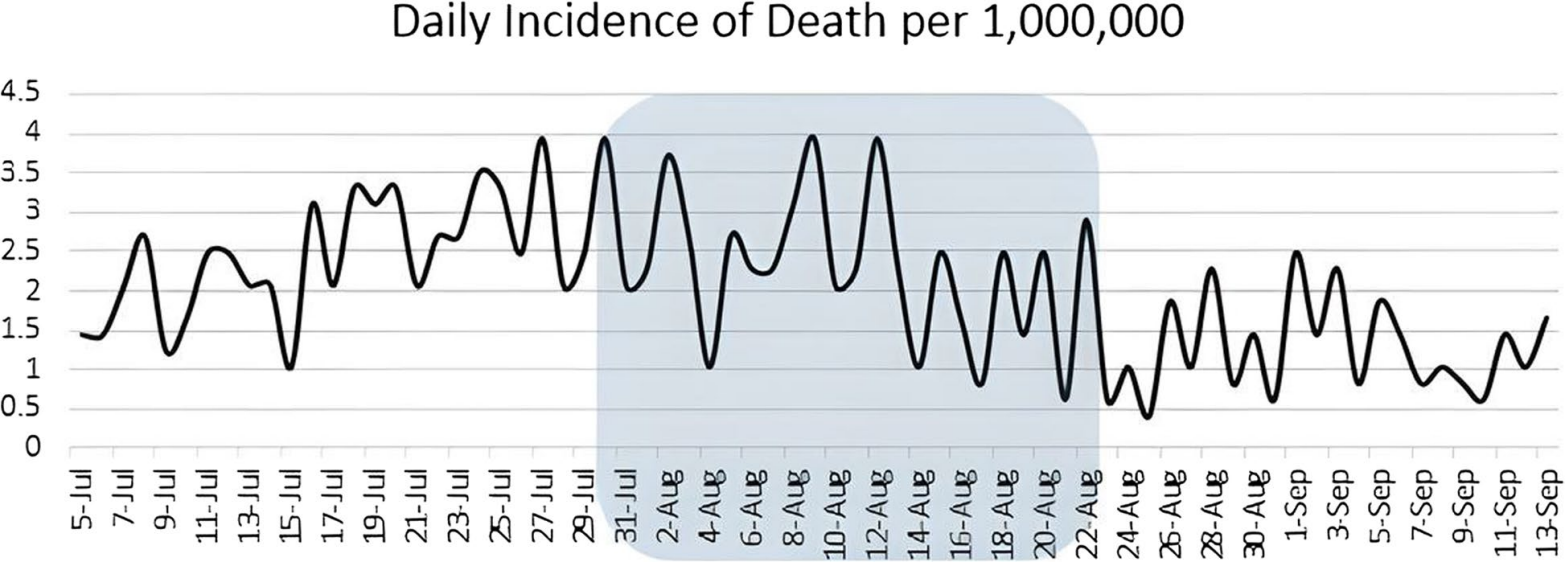
Daily number of deaths per 1,000,000. The square demonstrates the period in which NUEEs were conducted

Finally, our study evaluated the number of deaths from COVID-19. As it is depicted in Fig. 5, the daily number of deaths showed a decreasing pattern during the study period (coefficient: −0.02, *P* < 0.05).

## Discussion

It is necessary that policymakers establish their plans and decisions, specifically in regard to healthcare, based on evidence. NUEEs have been conducted during COVID-19 pandemic in some countries, while others decided to stop all of their in-person exams [16]. Since there had not been any studies regarding the effect of nationwide in-person exams, as a form of public gatherings, on the number of COVID-19 cases and its potential ramifications, this study aimed to evaluate the effects of NUEEs on COVID-19 related measurements and indices to create evidence for future similar conditions.

Our study revealed that conducting NUEEs during COVID-19 pandemic did not have any significant worsening effect on the trends of emergency services, hospital services, and death rates associated with COVID-19. The results of our study did not detect any increasing pattern in the number of newly confirmed COVID-19 cases following NUEEs during COVID-19 pandemic. Although there had not been similar studies in regard to the effect of conducting NUEEs or nationwide exams on the trend of COVID-19 pandemic, numerous studies assessed the effect of schools reopening or closure on COVID-19 pandemic. The majority of studies have shown that implementation of non-pharmacological interventions yields higher efficacy in limiting the spread of COVID-19, but the effects of schools reopening and closure on the pandemic have been controversial [17]. A recent study indicated that under strict hygiene measures combined with mandatory quarantines, schools reopening did not result in an increase in the number of COVID-19 cases [18]. Additionally, another study conducted in 21 Italian regions revealed that the reopening of schools did not lead to a second COVID-19 wave [19, 20].

On the other hand, a study conducted in the United States reported that school closure reduced mortality rates and led to a 62-percent weekly reduction in the number of new cases of COVID-19 [21]. Another study conducted by Miron O et al. indicated that the number of confirmed COVID-19 cases increased following schools reopening [18]. Our study, in a different context, did not find any associations between NUEEs and daily number of COVID-19 cases. We also witnessed a decreasing pattern in other indices and measurements related to COVID-19 (p-value for all indices and measurements was below 0.05). It is worthwhile to mention that the results of our study was against common expectations in which NUEEs, as mass public gatherings, are believed to induce a rise in the trends associated with COVID-19. This controversial result can be because of the discrepencies between the incidence rate of COVID-19 in total population compared to our NUEEs participants (0.63% in total population while 0.077% in NUEEs participants). One explanation is that the urgent nature of the exams encouraged individuals who were somehow involved with NUEEs to maintain minimum contact with other people in order to avoid contracting COVID-19. In addition, strict quarantine and containment rules and orders such as mandatory masks imposed by the government from July 5 to September 14, 2020 could have an additive impact on COVID-19 related trends and partially explain the decreasing trends in the number of new COVID-19 cases during this period.

SARS-CoV-2 can be transmitted through a variety of ways including coughing or sneezing within distance of less than 2 m and touching surfaces infected with the virus such as door handles, tables and chairs, faucets, staircase railings, and electrical switches [22]. Hence, strict health protocols play a huge role in COVID-19 containment. In Iran, the Ministry of Health prepared special health protocols for the conduct of NUEEs. These instructions included guidelines for exam participants and executive staff together with regulations for the exam centers. All parties involved were required to complete a self-declaration form before entering the exam venue stating that they did not have any respiratory signs and symptoms related to COVID-19 and that they had not been in close contact with any individuals with confirmed COVID-19 test. In the case of identifying suspects with positive signs, the separation of individuals and allocating them to a designated COVID-19 facility in a different building secluded from other participants was mandatory. Five confirmed COVID-19 cases whose information was recorded in the national Corona Lab system were isolated and took their university entrance exam at a designated COVID-19 facility. All exam venues were buildings that have rooms and halls with a capacity of at least 12 cubic meters with natural and industrial ventilation system. A minimum distance of 1.8 m between candidates, volunteers, and staff was implemented at all points of entry and exit and in all corridors, halls, and classrooms in the venue. The windows of the exam halls and corridors were kept open throughout the examination process. To protect the health and safety of the participants, the integrity of the protocols was rigourously monitored by ensuring that masks were used at all times, participants washed their hands for at least 20 s before the exam or disinfected their hands using hand sanitizers, and surfaces and tables were properly disinfected before and after the exams. When the exams were done and booklets were collected, candidates were asked to remain seated. The candidates were then ushered to separately leave the exam center while keeping appropriate social distancing [16].

Therefore, to enhance safety during health crises, it is suggested that policymakers prioritize evidence-based decision making and strengthen health protocols, including mandatory mask-wearing and social distancing. Implementing self-declaration forms for participants, establishing post-event surveillance for tracking COVID-19 cases, and launching public awareness campaigns on safety practices are essential.

### Limitations

The limitation of our study was that the evaluation of changes in the COVID-19 related measurements and indices after COVID-19 would have been more accurate if we had performed COVID-19 PCR test for all the participants of NUEEs. Nonetheless, we performed PCR test on individuals suspected to have COVID-19, but because of time and financial constraints, the urgent nature of COVID-19, lack of access to all participants, and failure to obtain permission from the ethics committee to conduct testing on all participants, we were not able to do so in mass on all NUEEs participants. Another limitation of this study was the failure to examine confounding factors at the individual level, which is one of the limitations of ecological studies that we encountered.

## Conclusion

This study has found that following the conduct of 2020 NUEEs in the province of Fars, there was no increasing trend in the number of COVID-19 cases and the associated indices and measurements. Considering the COVID-19 pandemic situation in Iran and its impacts on the educational and social systems, the results of this study are particularly significant. While some previous studies indicated an increase in COVID-19 cases due to large gatherings, our findings may indicate a positive impact from the implementation of strict health protocols. The findings of this study will be valuable for future practices, health protocols, and policies. However, since we are still scratching the surface with regard to pandemics, future research and studies on in-person nationwide exams and their impacts on different pandemics are recommended. This study also highlighted the importance of the constant implementation of strict health protocols and measures before, during, and after each exam.

## Data Availability

The datasets used and/or analysed during the current study are available from the corresponding author on reasonable request.

## Abbreviations

COVID-19: Coronavirus Disease 2019
WHO: World Health Organization
NUEEs: Nationwide University Entrance Exams
EMS: Emergency Medicine Services
PCR: Polymerase Chain Reaction
ICUs: Intensive Care Units
MCMC: Medical Care Monitoring
